# Telemedical Coaching Improves Long-Term Weight Loss in Overweight Persons: A Randomized Controlled Trial

**DOI:** 10.1155/2018/7530602

**Published:** 2018-09-09

**Authors:** Kerstin Kempf, Martin Röhling, Monika Stichert, Gabriele Fischer, Elke Boschem, Jürgen Könner, Stephan Martin

**Affiliations:** ^1^West-German Centre of Diabetes and Health, Düsseldorf Catholic Hospital Group, Düsseldorf, Germany; ^2^Occupational Health Services, Kassenärztliche Vereinigung Nordrhein, Düsseldorf, Germany; ^3^Occupational Health Services Ärztekammer Nordrhein, Düsseldorf, Germany; ^4^Occupational Health Services Eickhoff GmbH, Bochum, Germany

## Abstract

**Background:**

Lifestyle interventions have shown to be effective when continuous personal support was provided. However, there is lack of knowledge whether a telemedical-approach with personal coaching contributes to long-term weight losses in overweight employees. We, therefore, tested the hypothesis that telemedical-based lifestyle interventions accompanied with telemedical coaching lead to larger weight losses in overweight persons in an occupational health care setting.

**Methods:**

Overweight employees (n=180) with a body mass index (BMI) of >27 kg/m^2^ were randomized into either a telemedical (TM) group (n=61), a telemedical coaching (TMC) group (n=58), or a control group (n=61). Both intervention groups were equipped with scales and pedometers automatically transferring the data into a personalized online portal, which could be monitored from participants and coaches. Participants of the TMC group received additionally one motivational care call per week by mental coaches to discuss the current data (current weight and steps) and achieving goals such as a healthy lifestyle or weight reduction. The control group remained in routine care. Clinical and anthropometric data were determined after the 12-week intervention. Additionally, weight change was followed up after 12 months.

**Results:**

Participants of TMC (-3.1 ± 4.8 kg, p<0.0001) and TM group (-1.9 ± 4.0 kg; p=0.0012) significantly reduced weight and sustained it during the 1-year follow-up, while the control group showed no change. Compared to the control group only weight loss in the TMC group was significantly different (p<0.001) after 12 months. TMC and TM group also reduced BMI, waist circumference, and LDL cholesterol. Moreover, TMC group improved additionally systolic and diastolic blood pressure, total cholesterol, HDL cholesterol, and HbA1c.

**Conclusions:**

Telemedical devices in combination with telemedical coaching lead to significant long-term weight reductions in overweight persons in an occupational health care setting. This study is registered with NCT01868763, ClinicalTrials.gov.

## 1. Introduction

Positive energy balance and reduced physical activity are common reasons for weight gain [1]. Overweight and obesity not only increase the risk for several cardiometabolic diseases such as type 2 diabetes or coronary heart disease [1], but are also associated with sick leave days and increased disease costs [2]. Weight reduction and a healthy diet and a physically active lifestyle are generally recommended for overweight people to prevent type 2 diabetes [3, 4]. An analysis of the German socioeconomic panel data estimated costs of 2.5-5.4 billion EUR caused by overweight- and obesity-related sick leave days [2]. Therefore, companies should have an essential interest in effective health care programs for weight control of their employees. Accordingly, there is a strong need for effective lifestyle-based approaches and programs [5], particularly with psychosocial support [6]. Several worksite behavioral lifestyle interventions have shown to be feasible and effective in improving risk factors (e.g., weight loss, HbA1c) for diabetes and cardiovascular disease [7, 8]. Beneficial effects of lifestyle programs in an occupational health care setting comprise improvements in (i) absenteeism, (ii) productivity, and (iii) health care costs for employers [9, 10]. Therefore, lifestyle interventions have been successfully implemented in multiple community settings [11–14]. However, occupational health care settings have not been extensively examined [8, 15, 16]. In this context, telemedical and technology-based interventions comprise numerous advantages over traditional clinical settings such as convenience, cost, and the ability to tailor plans and feedback to a participant's individual needs. Nonetheless, telemedical interventions are facing certain problems such as absence of face-to-face interaction [17, 18]. Nevertheless, studies have already shown that telemedical coaching or telemonitoring can contribute to large reductions of body weight of more than 5% [19, 20]. However, the scientific discussion concerns the added value of telemedical coaching on telemonitoring alone [21, 22]. Nonetheless, in a previously published study, it has been shown that an additional personal support during a lifestyle intervention is essential and more effective in reducing weight and achieving goals than without human encouragement [23]. Continuous external feedback not only offers patients an additional contact person for medical questions regarding healthy diet and physical activity but also supports patients to further focus on their goals and stay motivated [24]. Furthermore, telephonically delivered lifestyle coaching interventions have been shown to support weight reduction and improve quality of life in different cohorts, even in patients with serious mental illnesses [19, 25].

In previous uncontrolled trials we had already evaluated the efficacy of a telemedical mental motivation program [26, 27], telemedically supported blood glucose self-monitoring [28, 29], and telemedical coaching [30]. However, there is still a lack of knowledge whether a telemedical-approach with in-person contact and personal coaching contributes to long-term weight losses and improvements in other cardiometabolic parameters.

We, therefore, tested in the present randomized controlled study the hypothesis that (i) a telemedical intervention with or without telemedical coaching leads to long-term weight losses and other beneficial clinical outcomes and (ii) whether telemedical coaching shows an additional impact on the results in overweight participants in an occupational health care setting.

## 2. Materials & Methods

### 2.1. Study Population

In an occupational health care setting overweight employees of the companies “*Gebr. Eickhoff Maschinenfabrik”*, the “*Ärztekammer Nordrhein”*, and the “*Kassenärztliche Vereinigung Nordrhein*” were invited by their medical corporate department for participation in the study. Eligible volunteers (n=180; inclusion criteria: 18-75 years old, body mass index (BMI) ≥ 27 kg/m^2^; exclusion criteria: acute diseases, severe illness with in-patient treatment during the last 3 months, weight reduction >2 kg/week during the last month, smoking secession during the last 3 months, drugs for active weight reduction, pregnancy, and breastfeeding) were randomized according to an electronically generated randomization list into three parallel groups. In detail, each participant was assigned a serial study identifier (ID). For each ID there was a closed envelope with the group assignment. The first participant was enrolled on 16.07.2012 and the last participant finished the study in 05.02.2014. The study was conducted at the West-German Centre of Diabetes and Health (WDGZ) in Düsseldorf, Germany, in accordance with the ethical standards laid down in the 1964 Declaration of Helsinki and its later amendments. The research protocol was approved by the ethics committee of the Ärztekammer Nordrhein, Düsseldorf, and was registered at clinicaltrials.gov under the number NCT01868763. All participants gave written informed consent prior to their inclusion into the study.

### 2.2. Study Design

Participants in the telemedical (TM) and telemedical coaching (TMC) group were equipped with telemetric scales (smartLAB scale W; HMM Holding AG, Dossenheim, Germany) and pedometer (smartLAB walk P+; HMM Holding AG, Dossenheim, Germany) automatically transferring recorded data into a personalized online portal. These data could be monitored from both, participant and the coaching team of the WDGZ. Participants in the TM group could monitor their body weight and steps (daily, weekly or average) but got no further support during the 12 weeks of intervention. The TMC group got additionally weekly care calls from trained coaches. These care calls included information about overweight or obesity-related diseases like type 2 diabetes, healthy diet, physical activity, and coping strategies for lifestyle changes. Moreover, acquired data were discussed (i.e., steps and weight) and participants were further motivated to achieve their individual goals (i.e., weight goals and healthy lifestyle changes) using a mental motivation program [26, 27] (Supplementary material). The control group remained in routine care. After the intervention phase telemetric devices remained in possession of the participants of both groups, and the participants were instructed to carry on measuring their weight and steps after the 12-week intervention period.

At baseline and after 12 weeks of intervention participants visited their medical corporate department for determination of anthropometric and clinical data (i.e., age, sex, body weight, height, BMI, waist circumference, blood pressure, total cholesterol, high-density lipoprotein (HDL) cholesterol, low-density lipoprotein (LDL) cholesterol, triglycerides, and hemoglobin A1c (HbA1c)). The assessors were blinded for group allocation. Body weight was measured in light clothing to the closest 0.1 kg, height to the closest 0.5 cm, and waist circumferences at the minimum abdominal girth (midway between the rib cage and the iliac crest). Blood pressure was measured after a five-minute rest in a sitting position on both arms. Venous blood was collected by inserting an intravenous cannula into the forearm vein and laboratory parameters were analyzed at the local laboratory. One year after the end of the intervention weight data out of the online portal were used for the follow-up analysis. These weight data were continuously recorded during the follow-up period and automatically transferred to the online portal by the scales. Afterwards, the online portal was closed after the 12-month follow-up.

### 2.3. Statistical Analysis

Primary endpoint was the reduction of body weight after 12 weeks of intervention and its later course during the 12-month follow-up compared between all of the three groups. Secondary endpoints were the changes in BMI, waist circumference, systolic and diastolic blood pressure, total cholesterol, HDL cholesterol, LDL cholesterol, triglycerides, and HbA1c after 12 weeks of intervention. Sample size had been calculated assuming that telemedical coaching might affect body weight. Our data indicated that due to telemedical lifestyle intervention a reduction of 2.3 kg in body weight in the TMC group can be assumed, while for the control group a reduction of only 1.0 kg was estimated. To be able to measure such a difference with a power of 90% and a level of significance of 5%, at least 50 datasets per group were needed. Since a dropout rate of 20% was estimated, the plan was to recruit 60 subjects per group, i.e., a total of 180 persons. Intention-to-treat analyses were performed. Missing values were substituted by the “last-observation-carried-forward” principle. Means ± standard deviations or standard error of means are shown, as appropriate. Baseline differences had been analyzed by using the Chi square test or Kruskal-Wallis test for nonparametric data and the ANOVA test for parametric data. The Wilcoxon signed-rank test was used for the analysis of differences within all the groups. The Kruskal-Wallis statistics with Dunn's multiple comparisons test was conducted for the comparison of Δ-values. The Friedman test with Dunn's multiple comparisons test was used to test the within group differences between time points. The Bonferroni correction was applied to adjust for multiple testing. Level of significance was set at p=0.05. Statistical analyses were performed using GraphPad Prism 6.04 (GraphPad Software, San Diego, CA, USA) and SAS statistical package version 9.3 (SAS Institute, Cary, NC, USA).

## 3. Results

### 3.1. Study Population

**Figure 1:**
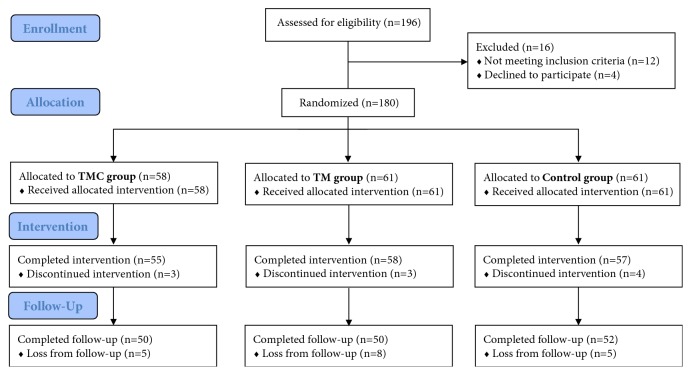
Flow diagram.

Fifty-eight participants were randomized to the TMC group, 61 to the TM group, and 61 to the control group (Figure 1). Baseline data did not differ significantly between all groups (Table 1). Distribution of BMI categories was also not different at baseline between all of the groups (Table 2). Fifty-five (95%) participants of TMC, 58 (95%) of TM, and 57 (93%) of the control group completed the 12-week intervention phase. Follow-up data after 12 months were available from 50 (86%), 50 (82%), and 52 (85%) participants, respectively. Main reasons for dropout were “personal/private reasons” and loss of motivation.

### 3.2. Weight Loss and Improvement of Cardiometabolic Risk Factors during the 12-Week Intervention

**Figure 2:**
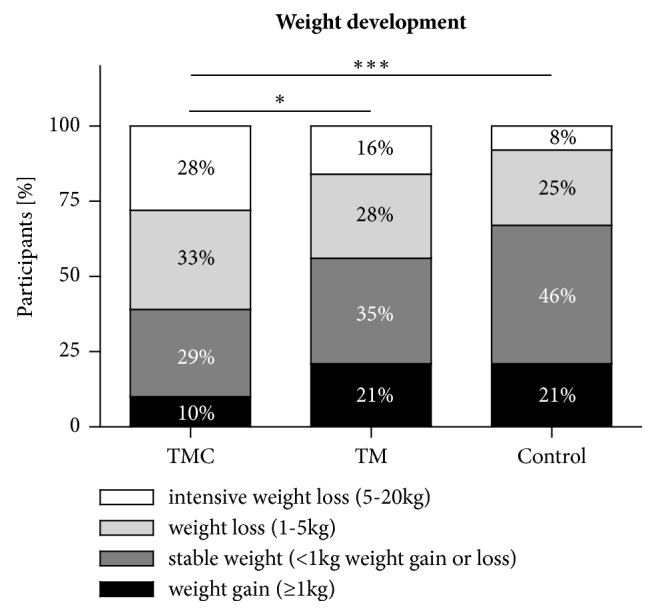
**Weight changes and differences after 12 weeks of intervention. **Participants of the telemedical coaching (TMC; n=58) group, the telemedical (TM; n=61) group, and the control group (n=61) were classified according to their weight change after 12 weeks of intervention into one of four categories: (1) ≥1 kg weight gain (black), (2) stable weight with <1 kg weight change (dark grey), (3) weight loss of 1-5 kg (light grey), or (4) weight loss of 5-20 kg (white). Shown are percentages. Differences in frequency distribution of weight change between the three groups were analyzed by using the Chi square test (*∗*, p<0.05; *∗∗∗*, p<0.001).

Participants of the TMC (98.9 ± 18.7 kg to 95.8 ± 16.9 kg (-3.1 ± 4.8 kg; p<0.0001) and the TM group (97.9 ± 17.4 kg to 96.0 ± 16.7 kg (-1.9 ± 4.0 kg; p=0.0012)) significantly reduced weight (Table 1). However, the weight of the control group remained statistically unchanged throughout the 12-weeks intervention. Compared to the control group, weight loss was only significant in the TMC group (p<0.001) after 12 weeks. The distribution of categories for weight changes differed significantly between groups (p=0.043 for TMC versus TM and p=0.0002 for TMC versus control; Figure 2). While the majority of participants in the TMC group achieved a weight loss, the proportion of participants with unchanged weight or weight gain was highest in the control group. Accordingly, a significant reduction of BMI was observed in the TMC group (p<0.0001) and in the TM group (p=0.0014). In comparison to the control group BMI reduction was only significant in the TMC group (p<0.001; Table 1). A significant reduction of waist circumference (p=0.0002 for the TMC, p=0.0033 for the TM, and p=0.0109 for the control group) was observed in all of the groups. However, the reduction in the control group did not remain statistically significant after correction for multiple testing. Moreover, cardiometabolic risk factors, i.e., systolic (p=0.039) and diastolic blood pressure (p=0.012), total cholesterol (p=0.014), HDL cholesterol (p=0.043), LDL cholesterol (p=0.006), and HbA1c (p=0.013) significantly improved in the TMC group, while only total cholesterol (p=0.013) and LDL cholesterol (p=0.006) improved in the TM group. Furthermore, proportion of persons with prediabetes (i.e., HbA1c: 5.7-6.4%) decreased by 7% in the TMC and by 6% in the TM group after 12 weeks of intervention.

### 3.3. Weight Change after 12 Months

**Figure 3:**
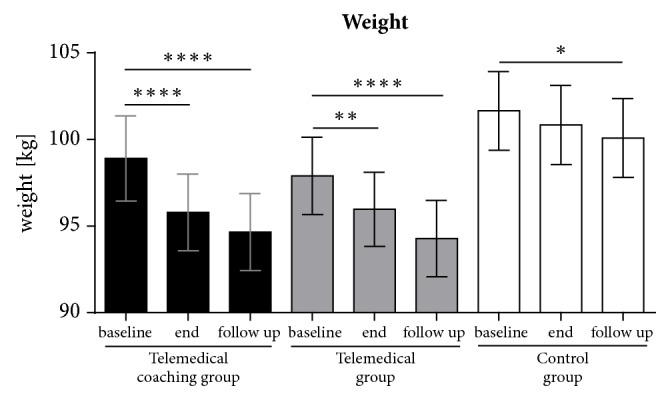
**Weight change and long-term effect.** Weight was determined at baseline, after 12 weeks of intervention and at the 52-week follow-up. Shown are means ± standard error of means. The Friedman test with Dunn's multiple comparisons test was used to test the within group differences between time points (*∗*, p<0.05; *∗∗*, p<0.01; *∗∗∗*, p<0.001; *∗∗∗∗*; p<0.0001).

Follow-up analysis of body weight demonstrated that participants of both intervention groups were further able to reduce weight after 12 months (Figure 3). In detail, participants of the TMC group further decreased their weight from 95.8 ± 16.9 kg to 94.7 ± 17.0 kg (-1.1 ± 2.4 kg; p<0.0001) in the period from week 12 to week 52. In sum, this represents a mean reduction of -4.2 ± 6.1 kg from baseline to week 52. The TM group decreased weight from 96.0 ± 16.7 kg to 94.3 ± 17.2 kg (-1.7 ± 4.8; p<0.0001) in the same study phase from week 12 to week 52. This represents a total reduction of 3.6 ± 6.1 kg from baseline to week 52. The control group showed also a slight decrease from 101.7 ± 17.7 kg to 100.1 ± 17.8 kg (-1.6 ± 3.5; p<0.05) after the 12-month follow-up.

## 4. Discussion

In the present randomized controlled three-armed study we could show that telemedical-based lifestyle interventions are applicable to motivate overweight individuals for lifestyle changes resulting in long-term weight reductions. In particular, the combination of telemedical devices and telemedical coaching led to greater reductions in body weight as well as improvements in cardiometabolic risk factors.

Other studies with different cohorts (e.g., persons with serious mental illness or obese patients with at least one cardiovascular risk factor) confirm our results and demonstrate that telemedical coaching or telemonitoring can contribute to relevant reductions of body weight of more than 5% [19, 20]. In particular, monitoring of physical activity (determined by accelerometers), body weight (daily recorded), and calorie intake (daily recorded) seems to be crucial for long-term (1-year period) weight management programs in obese patients, which is in line with our results [31]. Furthermore, the landmark study from Appel et al. investigated the effects of in-person support (face-to-face) in comparison to telemedical coaching (without face-to-face support) during a weight loss intervention program. Both lifestyle interventions achieved a meaningful weight reduction during a period of 24 months in obese patients. These important results underpin the potential of telemedical coaching and telemonitoring and indicate an effective solution for weight management support in the primary care and may be also for an occupational health care setting, even without face-to-face contact [20]. However, it has been shown that telephone calls alone, without telemedical coaching and monitoring, were not sufficient to sustainably influence behavior and reduce weight [21, 22] and demonstrated higher dropout rates as well [32]. In contrast, it has been shown due to a web-based weight management program that particularly the combination of automated web-based telemonitoring and basic nurse support (coaching) is an effective alternative for traditional weight management programs. The additional in-person support was essential for the weight reduction in comparison to the group without human encouragement. This relationship elucidates the necessity of external experts and coaching in telemedical interventions [23]. Moreover, Wi-Fi scales and other devices (e.g. smartphones or tablets with software applications) make it easier and more convenient for individuals to monitor their lifestyle, i.e. physical activity, diet, or weight measure. These behaviors are critical for short- and long-term weight control [33].

Besides the reduction of body weight, there were further relevant improvements in cardiovascular risk factors such as BMI, LDL cholesterol, and waist circumference in the present study. In line with other lifestyle intervention studies with electronic devices (web-, app- or SMS-based lifestyle interventions), external motivation, electronically transmitted reminders, or personalized coaching contribute to meaningful improvements in cardiovascular risk factors [20].

The present study was well tolerated. The overall dropout rate after 12 weeks of intervention and during the 12-month follow-up was 6% and 14%, and no adverse events were reported. This low dropout rate was also shown in patients with heart diseases during their cardiac rehabilitation (<10%) which was characterized by using telemedical devices (pedometers) accompanied by telephone coaching [34]. Possible explanations for these low dropout rates could be the flexible and easy contact with health experts as well as the more intense motivational coaching for lifestyle changes. Therefore, the number of telemedical lifestyle programs for the treatment of chronic diseases is increasing [35]. In light of this background, telemedical and telemonitoring channels (e.g., by call centers, internet-based programs, text messaging, or social networking sites) should improve their dissemination of intensive lifestyle programs to further improve the treatment of obesity. This development must be accompanied by far greater public health system efforts to prevent the development of obesity [33].

In a cohort of obese employees with a mean age of 47 years, 470-600 EUR additional costs per year for obesity-related sick leave days had been estimated [2]. Even with a conservative estimation, assuming constant costs, despite the increasing age and increasing number of comorbidities as well as early retirement, spending of 9.400-12.000 EUR per person during the next 20 years will arise. Since qualified telemedical coaching programs are available for less than 50 EUR per month [24] companies should rethink their current health care strategies and consider other options. Therefore, an external telemedical health care provider might be a cost-saving and promising alternative [36, 37]. The total for each patient in the present study was around 400 EUR including costs for equipment, coaching calls, and maintenance costs for the access to the online portal during the 12-week intervention phase. As the results of this trial are promising regarding weight control (4% weight reduction within 1 year), future occupational health care initiatives could use this treatment approach. When comparing the costs of the TMC program with the expanses for sick leave days [2], obesity-related drug costs [38], and considering the huge burden for the global or national health care systems [39, 40], one could argue that this telemedical treatment approach could be an efficient alternative. Furthermore, the possibility of repeating the program as well as the not-existing side-effects underline the usefulness of this treatment approach.

There are strengths and limitations in our study that should be mentioned. Overweight employees had been invited by their medical corporate department for participation in this study. Therefore, there could be a chance for a selection bias if only motivated employees agreed to participate. However, randomization into one of the three parallel groups should have abolished any potential effect, particularly, because baseline characteristics of the three groups were not different. On the other hand, a high motivation might have led to the low dropout rate of only 6% observed in our trial. According to the study size with 180 participants, the results of the present study might not be generalizable or transferable to other nonoccupational cohorts. In contrast to that, the study of Luley et al. demonstrated higher dropout rates of 9-12% during a 1-year lifestyle telemonitoring program for weight loss in obese patients with metabolic syndrome [31]. This difference could be the result of a less intense mentoring program with only monthly calls or weekly letters. Another limitation of the present study is the lack of data regarding diet of the participants during the study. Future studies should collect these data and analyze and follow up changes of eating behavior during and after the initial intervention phase. Furthermore, missing values were imputed by the LOCF approach in the present study. This procedure is a conservative method to estimate treatment effects of an intervention. Therefore, our results might have been underestimated by this approach, which should be considered when interpreting the data.

In sum, telemedical-based interventions are effective for long-term weight reductions in overweight employees. Especially in combination with continuous telephone coaching telemedical-based interventions demonstrated large effects on weight reduction and cardiovascular risk factors. These results underline the potential usefulness of telemedical monitoring and coaching for an occupational health care setting and could be an effective approach for preventive health care programs.

## Figures and Tables

**Table 1:** Study population characteristics.

	**TMC group (n=58)**			**TM group (n=61)**			**Control group (n=61)**		

Sex (male /female) [%]	52 / 48			39 / 61			44 / 56		

Age [years]	44 ± 10			45 ± 10			47 ± 10		

	**Baseline**	**End of intervention**	Δ	**Baseline**	**End of intervention**	Δ	**Baseline**	**End**	Δ

Weight [kg]	98.9 ± 18.7	**95.8 ± 16.9 ** **∗** **∗** **∗** **∗**	**-3.1 ± 4.8 ** ^##^	97.9 ± 17.4	**96.0 ± 16.7 ** **∗** **∗**	-1.9 ± 4.0	101.7 ± 17.7	100.8 ± 17.8	-0.8 ± 3.1

Body Mass Index [kg/m^2^]	32.7 ± 4.6	**31.7 ± 3.8 ** **∗** **∗** **∗** **∗**	**-1.0 ± 1.6 ** ^##^	32.9 ± 4.3	**32.3 ± 4.1 ** **∗** **∗**	-0.6 ± 1.3	34.0 ± 5.3	33.8 ± 5.4	-0.3 ± 1.0

Waist circumference [cm]	109 ± 12	**106 ± 10 ** **∗** **∗** **∗**	-3 ± 6	109 ± 13	**108 ± 13 ** **∗** **∗**	-2 ± 4	112 ± 13	111 ± 14 *∗*	-2 ± 5

Systolic blood pressure [mmHg]	141 ± 24	138 ± 24 *∗*	-4 ± 17	141 ± 23	139 ± 24	-3 ± 17	141 ± 17	140 ± 19	-1 ± 17

Diastolic blood pressure [mmHg]	90 ± 13	86 ± 12 *∗*	-4 ± 11	88 ± 14	88 ± 13	0 ± 10	89 ± 12	88 ± 11	-1 ± 12

Triglycerides [mg/dl] ^1^	191 ± 120	169 ± 94	-22 ± 77	167 ± 83	169 ± 153	-5 ± 115	191 ± 119	181 ± 127	-10 ± 62

Total cholesterol [mg/dl] ^1^	218 ± 33	207 ± 33 *∗*	-10 ± 28	210 ± 37	204 ± 36 *∗*	-5 ± 15	225 ± 44	226 ± 46	1 ± 22

HDL cholesterol [mg/dl] ^1^	51 ± 15	53 ± 15 *∗*	2 ± 7	51 ± 14	52 ± 13	1 ± 6	54 ± 18	55 ± 22	1 ± 11

LDL cholesterol [mg/dl] ^1^	135 ± 28	**130 ± 29 ** **∗** **∗**	-5 ± 22	131 ± 31	**126 ± 32 ** **∗** **∗**	-5 ± 14	140 ± 39	141 ± 41	1 ± 18

HbA1c [%] ^1^	5.6 ± 0.3	5.5 ± 0.3 *∗*	-0.1 ± 0.2	5.6 ± 0.3	5.5 ± 0.3	0.0 ± 0.1	5.7 ± 0.5	5.7 ± 0.4	0.0 ± 0.3

HbA1c 5.7-6.4% [%]	28	21	7	39	33	6	43	43	0

Shown are means ± standard deviations. Baseline differences had been analyzed by using the Chi square test and the ANOVA test. The Wilcoxon signed rank test was conducted for analysis of differences within all of the groups (*∗*, p<0.05; *∗∗*, p<0.01; *∗∗∗*, p<0.001; *∗∗∗∗*, p<0.0001). Kruskal-Wallis test with Dunn's multiple comparisons test was used to compare changes after 12 weeks of intervention between the intervention groups and the control group (^#^, p<0.05; ^##^, p<0.01; ^###^, p<0.001; ^####^, p<0.0001 compared to the control group). Bold written numbers indicate differences that remain statistically significant after Bonferroni correction for multiple testing. ^1^ missing values: n=6 in the telemedical coaching (TMC) group; n=3 in the telemedical (TM) and in the control group. HDL, high-density lipoprotein; LDL, low-density lipoprotein; HbA1c, hemoglobin A1C.

**Table 2:** Distribution of BMI categories between groups at baseline.

	**TMC group (n=58)**	**TM group (n=61)**	**Control group (n=61)**

**Overweight **(BMI <30 kg/m^2^) [n]	19 (32.8%)	17 (27.9%)	14 (23.0%)

**Moderately obese **(BMI 30-34,9 kg/m^2^) [n]	20 (34.5%)	26 (42.6%)	25 (41.0%)

**Severely obese **(BMI 35-39,9 kg/m^2^) [n]	16 (27.6%)	13 (21.3%)	13 (21.3%)

**Very severely obese** (BMI >40 kg/m^2^) [n]	3 (5.2%)	5 (8.2%)	9 (14.8%)

TMC, telemedical coaching group; TM, telemedical group. Frequency of BMI categories was not different between all groups.

## Data Availability

The data used to support the findings of this study are available from the corresponding author upon request.

## Supplementary Materials

Supplementary Materials The supplementary material contains (i) a general description of each telemedical coaching interview (pages 1 and 2) and (ii) general information about the conditions of the telemedical coaching (page 3).
