# Supplementary material for: Telemedical Coaching Improves Long-Term Weight Loss in Overweight Persons: A Randomized Controlled Trial
Source: Int J Telemed Appl. 2018 Sep 9;2018:7530602. doi: 10.1155/2018/7530602 (PMC6151211; doi:10.1155/2018/7530602)
Supplement: Supplementary Materials — The supplementary material contains (i) a general description of each telemedical coaching interview (pages 1 and 2) and (ii) general information about the conditions of the telemedical coaching (page 3). [file 7530602.f1.pdf]

Supplementary material.

<u>Content of telemedical coaching</u>

1. call/conversation

- Introductory conversation
- Explaining the study procedure to the participants
- Identification of personal resources
- Identification of personal motivations
- Medical history
- Anamnesis regarding physical activity
- Stress environment
- Occupational and daily routine
- Goal description (final/weekly/monthly)
- Next appointment

2. call/conversation

- Weekly goal achieved?
- Discussion about the transferred data (weight, steps)
- Concerns of the family doctor?
- Goal definition (weekly)
    - Physical activity
    - Diet (nutritional interview)
- Copying strategies for occupational and daily routine
- Next appointment

3. call/conversation

- Weekly goal achieved?
- Discussion about the transferred data (weight, steps) with additional regard to a healthy lifestyle comprising diet and increased physical activity.
- Main topic of call is motivation
- Nutritional anamnesis
- Goal definition (weekly)
    - Physical activity
    - Diet (nutritional interview)
- Next appointment

4.-5. call/conversation

- Weekly/monthly goals achieved?
- Discussion about the transferred data (weight, steps) with additional regard to a healthy lifestyle comprising diet and increased physical activity.

- Main topic of call is motivation for healthy lifestyle and its benefits
- Goal definition (weekly)
  - Physical activity
  - Diet (nutritional interview)
- Next appointment

6.-7. call/conversation

- Weekly/monthly goals achieved?
- Discussion about the transferred data (weight, steps) with additional regard to a healthy lifestyle comprising diet and increased physical activity.
- Main topic of call is dealing with setbacks
- Goal definition (weekly/monthly)
  - Physical activity
  - Diet (nutritional interview)
- Next appointment

8.-9. call/conversation

- Weekly/monthly goals achieved?
- Discussion about the transferred data (weight, steps) with additional regard to a healthy lifestyle comprising diet and increased physical activity.
- Main topic of call is dealing with setbacks
- Preparation for loss of contact to the coaches and self-initiated retention to the program
- Goal definition (weekly/monthly)
  - Physical activity
  - Diet (nutritional interview)
- Next appointment

10.-11. call/conversation

- Weekly/monthly goals achieved?
- Discussion about the transferred data (weight, steps) with additional regard to a healthy lifestyle comprising diet and increased physical activity.
- Main topic of call is dealing with setbacks and not achieving personal aims and reasons why
- Preparation for the time after the intervention phase
- Goal definition (monthly/year)
  - Physical activity
  - Diet (nutritional interview)
- Next appointment

12. call/conversation

- Final discussion

Coaching call checklists

Each coaching call checklist is prepared before each interview by the coaches depending on the content of each session. All telehealth coaches are trained in *Motivational Interviewing* and cognitive behavioral therapy techniques. The telehealth coaches help the participants to explore their internal motivation, discuss behavior change strategies as well as barriers to change, and answer any questions.

Coaching call appointments

Coaching call appointments are planned in accordance with the possibilities of the participants. Scheduled coaching calls are successfully completed, meaning the coach and participant complete a health coaching session. If a call was not completed, the missed call was dated at a time when participants were available.

Coaching platform

The coaches had access to an online platform where all data, regarding body weight and steps of the participants, were transferred to. These data were used as interview topics for each coaching call.
